# Impact of diabetes on COVID-19 and glucocorticoids on patients with COVID-19 and diabetes during the Omicron variant epidemic: a multicenter retrospective cohort study in South China

**DOI:** 10.1186/s12879-024-09287-z

**Published:** 2024-07-18

**Authors:** Yonghao Xu, Ling Sang, Ya Wang, Zhentu Li, Hongkai Wu, Xilong Deng, Nanshan Zhong, Xiaoqing Liu, Shiyue Li, Yimin Li

**Affiliations:** 1 Department of Critical Care Medicine, The First Affiliated Hospital of Guangzhou Medical University, Guangzhou Medical University, Guangzhou, China; 2 State Key Laboratory of Respiratory Disease, National Clinical Research Center for Respiratory Disease, Guangzhou Institute of Respiratory Health, First Affiliated Hospital of Guangzhou Medical University, Guangzhou Medical University, Guangzhou, China; 3 Guangzhou Laboratory, Guangzhou, China; 4 Department of Pulmonary and Critical Care Medicine, The First Affiliated Hospital of Guangzhou Medical University, Guangzhou, China; 5 Department of Critical Care Medicine, Guangzhou Eighth People’s Hospital, Guangzhou Medical University, Guangzhou, 510040 China

**Keywords:** Diabetes mellitus, Inflammation, SARS-CoV-2, Glucocorticoids

## Abstract

**Background:**

To explore the impact of diabetes on the clinical features and prognosis of COVID-19 and assess the influence of glucocorticoid use on the prognosis of patients with COVID-19 and diabetes.

**Methods:**

This retrospective multicenter cohort study included patients admitted between December 2022 and January 2023. The patients were grouped according to diabetes and glucocorticoid use. The primary outcome was in-hospital mortality.

**Results:**

Among 400 patients with glucocorticoid data, 109 (27.3%) had diabetes. The inflammatory cytokines were higher in patients with diabetes, manifested by higher IL-6 (25.33 vs. 11.29 ng/L, *p* = 0.011), CRP (26.55 vs. 8.62 mg/L, *p* = 0.003), and PCT (0.07 vs. 0.04 ng/ml, *p* = 0.010), while CD4^+^ (319 vs. 506 /mL, *p* = 0.004) and CD8^+^ (141 vs. 261 /mL, *p* < 0.001) T lymphocytes were lower. The overall mortality rate of hospitalized COVID-19 patients with diabetes was 13.46%. The diabetic patients who received glucocorticoids vs. those who did not receive glucocorticoids had a similar mortality (15.00% vs. 11.39%, *p* = 0.591).

**Conclusions:**

Patients with COVID-19 and diabetes are more likely to experience hyperinflammatory response and T cell reduction, especially those with severe/critical disease. Glucocorticoid use was not associated with the prognosis of COVID-19 in patients with diabetes. Still, glucocorticoids should be used cautiously in diabetic patients with severe/critical COVID-19.

**Supplementary Information:**

The online version contains supplementary material available at 10.1186/s12879-024-09287-z.

## Background

After 3 years of the pandemic, the coronavirus disease 2019 (COVID-19) is still spreading worldwide [1]. The Omicron variant has gradually become a major strain in China since early 2022 [2, 3]. Despite the general opinion that the pathogenicity of the Omicron variant is lower than its predecessors because its major manifestations are in the upper respiratory system, patients with severe COVID-19 can still be observed in special populations, and such patients have a poor prognosis [4–6]. Indeed, about 24% of patients hospitalized for COVID-19 have at least one comorbidity [7].

Among the common morbidities, diabetes is a risk factor for swift COVID-19 progression and poor prognosis [8, 9]. Indeed, diabetes is a pro-inflammatory condition often accompanied by immune dysfunction [10, 11]. Patients with diabetes can present a state of hyperinflammation and immunosuppression when they are infected with SARS-CoV-2 [8]. Thus, examining the impact of Omicron variant infection in patients with diabetes is necessary because of the differences in the infection characteristics compared with previous SARS-CoV-2 variants.

Glucocorticoids are a treatment option for patients with severe COVID-19 [12–16]. Indeed, several studies demonstrated the beneficial effects of glucocorticoids on patients hospitalized for COVID-19, and the underlying mechanisms could involve the inhibitory effects of glucocorticoids on the inflammatory response [12–16]. The World Health Organization (WHO) guidelines for the clinical management of COVID-19 recommend corticosteroids for severe and critical COVID-19 patients [17]. China’s Strategies for Diagnosis and Treatment of SARS-CoV-2 Infection (Trial Version 10) [18] also indicates that glucocorticoids could be used for severe and critical cases when necessary. On the other hand, glucocorticoids can elevate the blood glucose levels in patients with COVID-19 [19], leading to complications and the risk of poorer outcomes in patients with diabetes. The impact of glucocorticoids in patients with diabetes and the Omicron variant COVID-19 is unknown.

Therefore, this study explored the impact of diabetes on the clinical features and prognosis of COVID-19 and assessed the influence of glucocorticoid use on the prognosis of patients with diabetes and the Omicron variant COVID-19. The results could help guide the management of COVID-19 in the special population of patients with diabetes.

## Methods

### Study design and patients

This retrospective multicenter cohort study included patients admitted between December 8, 2022, and January 19, 2023, at the First Affiliated Hospital of Guangzhou Medical University, the Second Affiliated Hospital of Guangzhou Medical University, the Third Affiliated Hospital of Guangzhou Medical University, the Fourth Affiliated Hospital of Guangzhou Medical University, Guangzhou Eighth People’s Hospital, and the Affiliated Cancer Hospital and Institute of Guangzhou Medical University. This study was approved by the Ethics Committee of the First Affiliated Hospital of Guangzhou Medical University as the lead center (approval #ES-2023-015-01) and by the ethics committees of the other sites. The Ethics Committee of the First Affiliated Hospital of Guangzhou Medical University waived the requirement for informed consent due to the retrospective nature of the study.

The inclusion criteria were (1) adult patients (> 18 years of age) previously diagnosed with diabetes, (2) a positive laboratory test for SARS-CoV-2 for a respiratory tract or blood sample by reverse transcription polymerase chain reaction (RT-PCR), and (3) a positive antigen test for SARS-CoV-2 from a respiratory tract sample. The exclusion criteria were (1) incomplete data regarding corticosteroids (e.g., time, dose, or categories) or (2) hospital stay < 48 h.

The disease was classified as mild, moderate, severe, and critical according to China’s Strategies for Diagnosis and Treatment of SARS-CoV-2 Infection (Trial Version 10) [18]. (1) Mild: upper respiratory tract infection as the main manifestation, with symptoms such as pharyngeal discomfort, cough, and fever. (2) Moderate: persistent high fever for > 3 days, and/or symptoms such as cough or shortness of breath, but respiratory rate < 30 breaths/min, and oxygen saturation > 93% on room air at rest; chest imaging showing the characteristic manifestations of SARS-CoV-2 pneumonia. (3) Severe: at least one of the following conditions that cannot be explained by other reasons: (i) shortness of breath and respiratory rate of ≥ 30 breaths/min, (ii) oxygen saturation of ≤ 93% on room air at rest, and/or (iii) arterial partial pressure of oxygen (PaO_2_)/oxygen uptake concentration (FiO_2_) ratio of ≤ 300 mmHg. 4) Critical: at least one of the following conditions: (i) mechanical ventilation required because of respiratory failure, (ii) shock, and/or (iii) transfer to the ICU required because of other organ failure.

The diagnosis of diabetes was based on the presence of a previous clinical diagnosis of diabetes, made based on the American Diabetes Association (ADA2022) guidelines for diabetes [20]. Patients who visited other medical facilities prior to hospitalization but had no clear records of the use of glucocorticoids were excluded. Pregnant women were also excluded. The patients were divided into the diabetic and non-diabetic groups. The patients with diabetes were further divided according to glucocorticoid use.

### Outcomes and data collection

The cross-section of data collection was from 2023-01-20 until the last patient reaches the final outcome of hospitalization (death or discharge). A webpage reports that the main circulating strains in China as of January 2023 are BA.5.2 and BF.7 (World Health Organization: TAG-VE statement on the meeting of 3 January on the COVID-19 situation in China [21]. The primary outcome was in-hospital mortality. Follow-up was censored on February 28, 2023. All patient data were collected from the hospital information system at each center, including demographic and clinical characteristics, clinical laboratory findings, previous medical history, complications, vaccination status, and the use of glucocorticoids. A standardized data collection form with clear criteria for recording both categorical and continuous variables was used by all participating centers. All collected data were checked by two investigators who did not participate in the data collection.

The patients treated with intravenous or oral glucocorticoids, including dexamethasone, prednisone, prednisolone, methylprednisolone, and hydrocortisone, during COVID-19 disease duration (treatments before and after hospitalization) were considered treated with glucocorticoids.

Age, gender, body mass index (BMI), smoking history, vaccination, and comorbidities (including diabetes) were collected at admission for each patient. Blood glucose, COVID-19 severity, glucocorticoids, treatments, vital signs, and blood gas analysis were collected at admission and during hospitalization. Length of hospitalization and in-hospital mortality were assessed during hospitalization. All these laboratory tests are performed in different hospital laboratories and we did not collect patient specimens and use the same laboratory kits for testing.

### Statistical analysis

R 4.2.1 was used for statistical analysis. Continuous data with a normal distribution (according to the Kolmogorov-Smirnov test) were described as means ± standard deviations and analyzed using Student’s t-test; otherwise, they were presented as medians (interquartile range (IQR)) and analyzed using the Wilcoxon rank-sum test. Categorical data were described as n (%) and analyzed using the chi-square test or Fisher’s exact test. The factors independently associated with mortality were identified using a multivariable logistic regression analysis. Factors with *P*-values < 0.05 in the univariable analyses were included in the multivariable analysis. Survival was analyzed using the Kaplan-Meier method, and the curves were compared using the log-rank test. Two-sided *p*-values < 0.05 were considered statistically significant.

## Results

### Baseline data and demographic characteristics

**Fig. 1:**
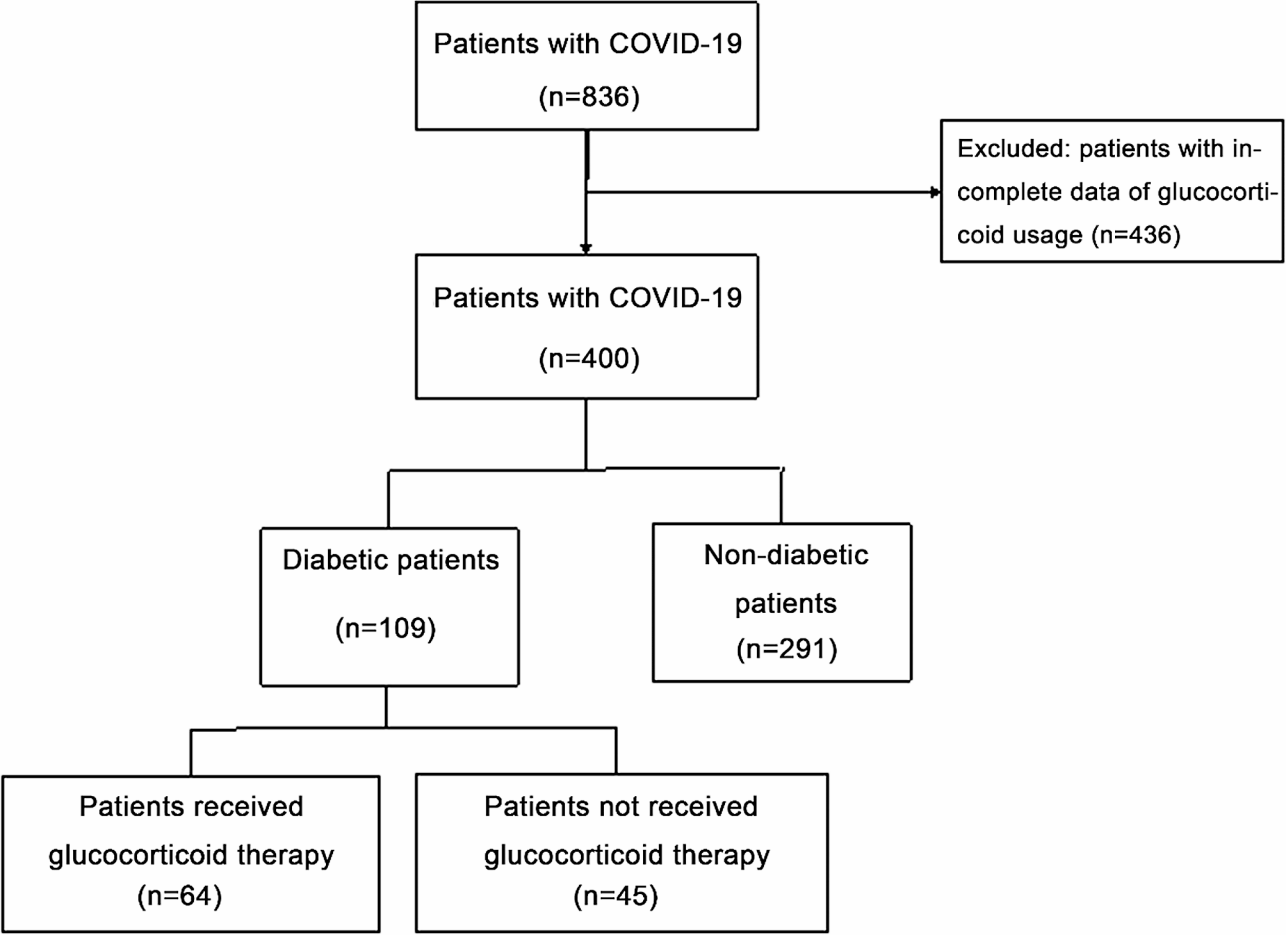
Flowchart of patient inclusion

In this study, 836 patients with COVID-19 met the inclusion and exclusion criteria, but only 400 patients had complete and definite data about glucocorticoids were included for analysis. The severity classification and incidence of clinical symptoms were showed in Table S1. Among the 400 patients, 109 (27.3%) had diabetes. In the 109 patients with diabetes, 6 (5.5%) had mild COVID-19, 39 (35.78%) were moderate, 33 (30.28%) were severe, and 31 (28.44%) were critical. While, in the other 291 non-diabetic patients, 52 (17.87%) were mild, 125 (42.96%) were moderate, 42 (14.43%) were severe, and 72 (24.74%) were critical. Among diabetic patients, 64 received glucocorticoids (Fig. 1). Compared with the non-diabetes group, the patients in the diabetes group were older (74 vs. 69 years, *p* < 0.001), had a higher body mass index (BMI) (23.42 vs. 22.88 kg/m^2^, *p* = 0.030), higher frequencies of severe/critical COVID-19 (58.72% vs. 39.18%, *p* < 0.001), cardiovascular diseases (82.57% vs. 44.33%, *p* < 0.001), and chronic kidney diseases (16.82% vs. 7.69%, *p* = 0.008), and a lower frequency of chronic pulmonary diseases (14.95% vs. 24.83%, *p* = 0.036) (Table 1). The oxyhemoglobin saturation (96% vs. 97%, *p* = 0.012) and oxygen partial pressure (85.3 vs. 93.7 mmHg, *p* = 0.080) were low in both groups when the concentration of oxygen inhalation was similar. The frequency of patients requiring continuous renal replacement therapy (CRRT) was higher in the diabetic group than in the non-diabetic group (6.60% vs. 1.75%, *p* = 0.032). Regarding antiviral therapy, the proportions of patients who received nirmatrelvir/ritonavir, azvudine, or neutralizing antibody (ambavimab/romisivirmab) were not significantly different between the diabetic and non-diabetic groups (Table 2).

**Table 1:** Basic demographic characteristics of the patients

Variable	Total (*n* = 400)	Diabetic group (*n* = 109)	Non-diabetic group (*n* = 291)	*P* value
Age (years)	71 (60–81) (*n* = 400)	74 (67–83) (*n* = 109)	69 (58–80) (*n* = 291)	< 0.001
Gender				
Male	244/400 (61.00)	64/109 (58.72)	180/291 (61.86)	0.567
Female	156/400 (39.00)	45/109 (41.28)	111/291 (38.14)	
Body mass index (kg/m^2^)	23.08 (20.43–25.39) (*n* = 340)	23.42 (21.58–26.10) (*n* = 96)	22.88 (20.02–25.26) (*n* = 244)	0.030
In-hospital mortality	47/384 (12.24)	14/104 (13.46)	33/280 (11.79)	0.656
Smoking history	96/391 (24.55)	24/106 (22.64)	72/285 (25.26)	0.592
Time from disease onset to hospitalization (d)	9 (5–13) (*n* = 399)	9 (4–12) (*n* = 109)	9 (5–14) (*n* = 290)	0.803
Clinical type				< 0.001
Severe/critical	178/400 (44.50)	64/109 (58.72)	114/291 (39.18)	
Mild/moderate	222/400 (55.50)	45/109 (41.28)	177/291 (60.82)	
Vaccination				0.813
Not vaccinated	154/383 (40.21)	49/107 (45.79)	105/276 (38.04)	
1 dose	23/383 (6.01)	9/107 (8.41)	14/276 (5.07)	
2 doses	58/383 (15.14)	17/107 (15.89)	41/276 (14.86)	
3 doses or more	148/383 (38.64)	32/107 (29.91)	116/276 (42.03)	
Comorbidities				
Cardiovascular disease	219/400 (54.75)	90/109 (82.57)	129/291 (44.33)	< 0.001
Chronic pulmonary disease	87/393 (22.14)	16/107 (14.95)	71/286 (24.83)	0.036
Chronic kidney disease	40/393 (10.18)	18/107 (16.82)	22/286 (7.69)	0.008
Chronic liver disease	16/393 (4.07)	4/107 (3.74)	12/286 (4.20)	> 0.999
Others	85/363 (23.42)	17/97 (17.53)	68/266 (25.56)	0.110
At least one comorbidity	334/393 (84.99)	109/109 (100.00)	225/284 (79.23)	< 0.001

**Table 2:** Treatments during hospitalization

Variable	Total (*n* = 400)	Diabetic group (*n* = 109)	Non-diabetic group (*n* = 291)	*P* value
Glucocorticoid therapy during hospitalization	214/400 (53.50)	64/109 (58.72)	150/291 (51.55)	0.201
Nirmatrelvir/ritonavir	91/391 (23.27)	25/106 (23.58)	66/285 (23.16)	0.929
Azvudine	11/391 (2.81)	2/106 (1.89)	9/285 (3.16)	0.740
Neutralizing antibody	14/391 (3.58)	7/106 (6.60)	7/285 (2.46)	0.098
Continuous renal replacement therapy	12/391 (3.07)	7/106 (6.60)	5/285 (1.75)	0.032
Extracorporeal membrane oxygenation	0/391 (0.00)	0/106 (0.00)	0/285 (0.00)	> 0.999
Intra-aortic balloon pump	0/391 (0.00)	0/106 (0.00)	0/285 (0.00)	> 0.999

Neutralizing antibody: ambavimab/romisivirmab

### Prognosis of patients with diabetes and COVID-19

**Fig. 2:**
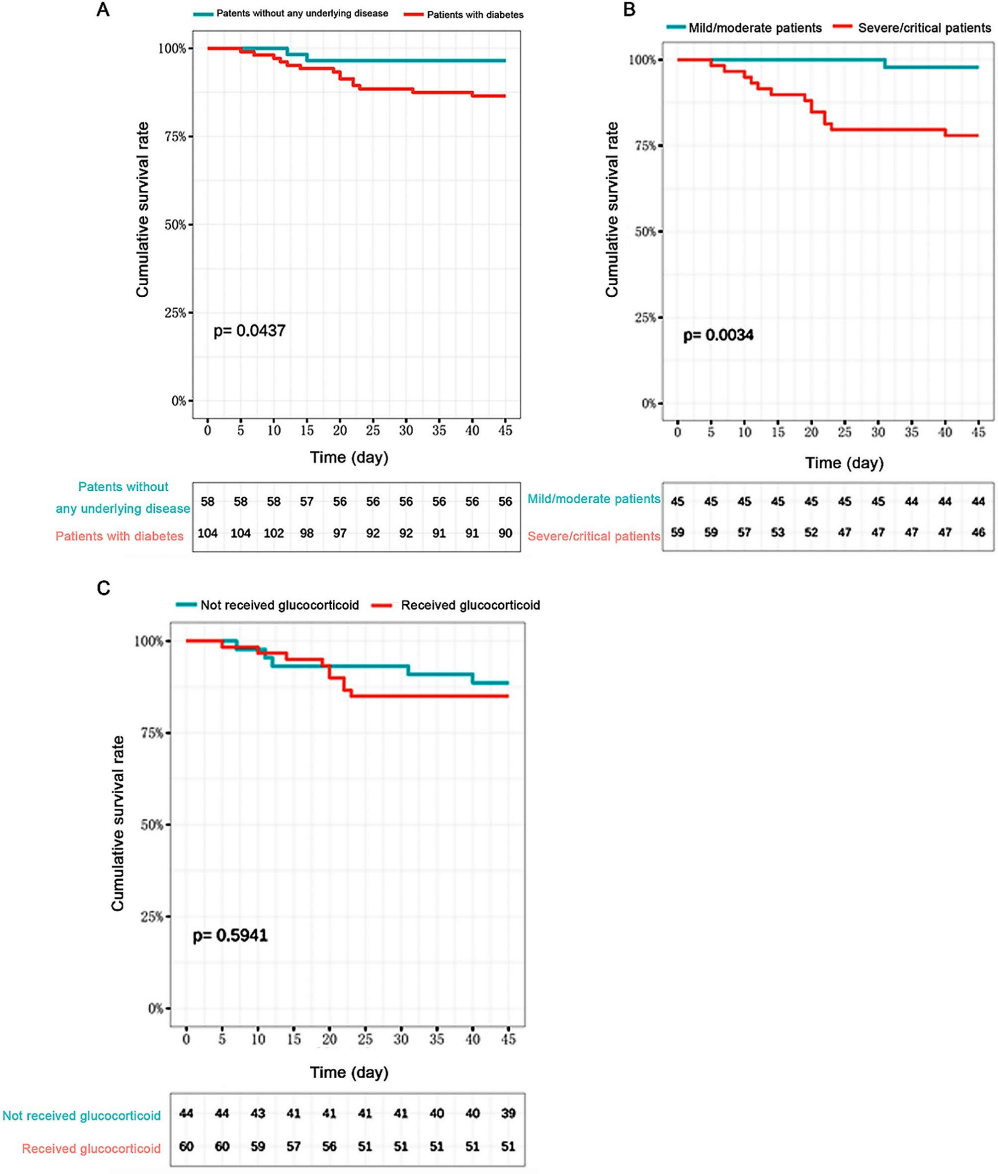
Kaplan-Meier survival curves. **(A)** Survival curves of patients with diabetes and those without any comorbidity. **(B)** Survival curves of mild/moderate and severe/critical patients. **(C)** Survival curves of patients who received glucocorticoids and those who did not

There were 384 patients with 45 days of follow-up data (104 with diabetes and 280 without). Compared with the patients without any comorbidity (*n* = 58), the mortality risk of the patients with diabetes (104 cases) was significantly higher (*p* = 0.044) (Fig. 2a). The mortality rate of patients with severe/critical COVID-19 was also significantly higher than in patients with mild/moderate COVID-19 (*p* = 0.003) (Fig. 2b). Multivariate logistic regression analysis showed that severe/critical clinical type (OR = 12.49, 95%CI: 2.19-237.94, *P* = 0.020) were independently associated with survival in patients with diabetes and COVID-19 (Table 3).

**Table 3:** Multivariable logistic regression analysis of independent predictors of survival in diabetic patients

	All (*n* = 104)	Died (*n* = 14)	Survived (*n* = 90)	*P*	Multivariable logistic regression		
					OR (95% CI)		*P*
Age (years)	74 (66–82)	83 (72–87)	74 (66–82)	0.034		1.08 (1.00-1.17)	0.058
Gender							
Male	60/104 (57.7)	8/14 (57.1)	52/90 (57.8)	0.964			
Female	44/104 (42.3)	6/14 (42.9)	38/90 (42.2)	0.964			
Body mass index	23.44 (21.55–26.15) (*n* = 91)	23.40 (22.22–29.05) (*n* = 12)	23.44 (21.47–25.72) (*n* = 79)	0.231			
Smoking	21/101 (20.8)	1/13 (7.7)	20/88 (22.7)	0.378			
Time from disease onset to hospitalization (d)	9 (4–12)	9 (4–10)	9 (4–12)	0.928			
Vaccination							
No vaccination	45/102 (44.1)	10/14 (71.4)	35/88 (39.8)	0.027		2.56 (0.71–10.59)	0.163
1 dose	9/102 (8.8)	0/14 (0.0)	9/88 (10.2)	0.456			
2 doses	16/102 (15.7)	2/14 (14.3)	14/88 (15.9)	1.000			
3 doses	32/102 (31.4)	2/14 (14.3)	30/88 (34.1)	0.241			
Clinical type				0.003		12.49 (2.19-237.94)	0.020
Severe/critical	59/104 (56.7)	13/14 (92.9)	46/90 (51.1)				
Mild/moderate	45/104 (43.3)	1/14 (7.1)	44/90 (48.9)				
Comorbidities							
Cardiovascular disease	86/104 (82.7)	12/14 (85.7)	74/90 (82.2)	1.000			
Chronic pulmonary disease	16/102 (15.7)	1/13 (7.7)	15/89 (16.9)	0.660			
Chronic kidney disease	17/102 (16.7)	2/13 (15.4)	15/89 (16.9)	1.000			
Chronic liver disease	4/102 (3.9)	0/13 (0.0)	4/89 (4.5)	1.000			
Others	16/94 (17.0)	3/10 (30.0)	13/84 (15.5)	0.478			
At least one comorbidity	104/104 (100.0)	14/14 (100.0)	90/90 (100.0)	1.000			
Glucocorticoids used during hospitalization	60/104 (57.7)	9/14 (64.3)	51/90 (56.7)	0.591			

### Immune and inflammatory status of the patients with vs. without diabetes

Compared with the non-diabetic group, the diabetic group had significantly higher levels of inflammatory factors, including interleukin (IL)-6 (25.33 vs. 11.29 ng/L, *p* = 0.011), C-reactive protein (CRP) (26.55 vs. 8.62 mg/L, *p* = 0.003), and procalcitonin (PCT) (0.07 vs. 0.04 ng/mL, *p* = 0.010). The levels of CD4^+^ T lymphocytes (319 vs. 506 /ml, *p* = 0.004) and CD8^+^ T lymphocytes (141 vs. 261 /ml, *p* < 0.001) were low in the diabetic group compared with the non-diabetic group, reflecting that the immune functions were low in the diabetic group and suggesting a risk of hyperinflammation and T cell immunosuppression (Table 4).

**Table 4:** Clinical characteristics of patients with COVID-19.

Variable	All (*n* = 400)	Diabetic group (*n* = 109)	Non-diabetic group (*n* = 291)	*P* value
Vital signs on hospital admission				
Body temperature (°C)	36.5 (36.3–36.8) (*n* = 387)	36.5 (36.3–36.8) (*n* = 106)	36.5 (36.3–36.8) (*n* = 281)	0.211
Respiration rate (times/min)	21 (20–22) (*n* = 387)	21 (20–22) (*n* = 106)	21 (20–22) (*n* = 281)	0.868
Heart rate (beats/min)	88 (79–100) (*n* = 386)	85 (77–98) (*n* = 105)	89 (80–101) (*n* = 281)	0.016
Systolic pressure (mmHg)	125 (116–140) (*n* = 386)	129 (115–144) (*n* = 106)	125 (117–137) (*n* = 280)	0.178
Diastolic pressure (mmHg)	76 (69–84) (*n* = 385)	75 (68–83) (*n* = 105)	77 (70–84) (*n* = 280)	0.197
Blood-gas analysis on hospital admission				
SPO_2_ (%)	97.0 (95.0–98.0) (*n* = 332)	96.0 (93.0–98.0) (*n* = 96)	97.0 (95.0–98.0) (*n* = 236)	0.011
pH	7.402 (7.364–7.437) (*n* = 358)	7.403 (7.361–7.441) (*n* = 103)	7.400 (7.365–7.435) (*n* = 255)	0.777
PaO_2_ (mmHg)	91.9 (73.5–129.0) (*n* = 357)	85.3 (69.3-114.2) (*n* = 102)	93.7 (75.4-133.3) (*n* = 255)	0.080
PaCO_2_ (mmHg)	36.2 (32.0-41.5) (*n* = 357)	35.9 (32.0-39.3) (*n* = 102)	37.0 (32.2–43.1) (*n* = 255)	0.015
FiO_2_ (%)	21 (21–29) (*n* = 323)	21 (21–29) (*n* = 91)	21 (21–29) (*n* = 232)	0.802
HCO_3_^−^ (mmol/L)	23.1 (21.1–25.2) (*n* = 351)	22.6 (19.6–24.7) (*n* = 100)	23.2 (21.5–25.4) (*n* = 251)	0.027
First measurements of biochemical indicators after hospitalization				
White blood cell count (10^9^/L)	7.35 (5.53–10.69) (*n* = 376)	7.39 (5.55–11.47) (*n* = 105)	7.32 (5.52–10.62) (*n* = 271)	0.648
Lymphocyte count (10^9^/L)	1.05 (0.60–1.60) (*n* = 375)	0.94 (0.60–1.38) (*n* = 105)	1.10 (0.63–1.70) (*n* = 270)	0.053
Platelet count (10^9^/L)	229 (171–301) (*n* = 374)	208 (147–302) (*n* = 104)	235 (179–300) (*n* = 270)	0.125
Na^+^ (mmol/L)	137.4 (134.3-140.1) (*n* = 363)	136.4 (134.1–139.0) (*n* = 101)	137.9 (134.6-140.2) (*n* = 262)	0.068
K^+^ (mmol/L)	3.87 (3.57–4.20) (*n* = 363)	3.95 (3.61–4.29) (*n* = 101)	3.83 (3.57–4.15) (*n* = 262)	0.029
Cl^−^ (mmol/L)	103.9 (99.8-106.7) (*n* = 362)	102.2 (99.4-107.9) (*n* = 101)	104.1 (100.2-106.3) (*n* = 261)	0.830
Blood glucose (mmol/L)	7.46 (5.60-10.29) (*n* = 357)	11.47 (7.91-15.00) (*n* = 99)	6.75 (5.37–8.69) (*n* = 258)	< 0.001
D-dimer (ng/mL) (ref: <500)	831 (467–1447) (*n* = 369)	920 (606–1493) (*n* = 104)	745 (410–1397) (*n* = 265)	0.040
CRP (mg/L) (ref: <0.6)	10.70 (3.00-52.26) (*n* = 230)	26.55 (5.83–84.51) (*n* = 70)	8.62 (2.07–47.40) (*n* = 160)	0.003
PCT (ng/ml) (ref: <0.05)	0.04 (0.02–0.25) (*n* = 342)	0.07 (0.03–0.38) (*n* = 97)	0.04 (0.02–0.23) (*n* = 245)	0.010
Ferritin (ng/mL) (ref: 30–400)	838 (442–1376) (*n* = 152)	910 (537–1600) (*n* = 52)	783 (357–1233) (*n* = 100)	0.137
ESR (mm/h) (ref: <15)	52 (28–78) (*n* = 291)	64 (40–92) (*n* = 82)	46 (24–71) (*n* = 209)	0.002
LDH (U/L) (ref: 120–250)	262.8 (203.7-339.2) (*n* = 328)	264.1 (215.8–361.0) (*n* = 88)	262.2 (197.5-331.2) (*n* = 240)	0.357
Troponin/hypersensitive troponin (ng/ml) (ref: <17.5)	7.70 (4.30-15.89) (*n* = 310)	11.00 (5.70–19.50) (*n* = 87)	6.50 (3.90–12.90) (*n* = 223)	< 0.001
Myoglobin (ng/ml) (ref: <70)	45.6 (26.0-94.9) (*n* = 342)	68.4 (37.9-143.1) (*n* = 94)	40.8 (24.1–90.4) (*n* = 248)	< 0.001
Creatinine (µmol/L) (ref: 65.4-119.3)	85.3 (71.8-107.4) (*n* = 363)	93.2 (74.3-125.3) (*n* = 102)	83.2 (71.4–99.9) (*n* = 261)	0.010
Urea nitrogen (mmol/L) (ref: 3.1-8.0)	6.5 (4.7–9.6) (*n* = 363)	8.4 (5.5–12.4) (*n* = 101)	6.0 (4.4–8.3) (*n* = 262)	< 0.001
ALT (U/L) (ref: 9–50)	23.2 (15.6–35.4) (*n* = 323)	23.4 (15.9–37.2) (*n* = 87)	23.1 (15.4–34.8) (*n* = 236)	0.572
First measurements of cytokines and immune cell count after hospitalization				
IL-6 (ng/L) (ref: <17.4)	12.44 (4.18–41.25) (*n* = 227)	25.33 (8.36–47.64) (*n* = 61)	11.29 (3.76–36.82) (*n* = 166)	0.011
IL-8 (ng/L) (ref: <20.6)	52.00 (25.40-124.66) (*n* = 170)	48.05 (27.12-144.76) (*n* = 43)	54.59 (25.24-124.23) (*n* = 127)	0.902
IFN-r (ng/L) (ref: <7.42)	0.93 (0.28–1.74) (*n* = 191)	1.36 (0.40–2.19) (*n* = 51)	0.90 (0.28–1.60) (*n* = 140)	0.063
CD4^+^ T cells (/ml) (ref: 550–1440)	462 (203–693) (*n* = 142)	319 (129–574) (*n* = 38)	506 (308–825) (*n* = 104)	0.004
CD8^+^ T cells (/ml) (ref: 320–1250)	238 (124–387) (*n* = 133)	141 (43–256) (*n* = 37)	261 (150–438) (*n* = 96)	0.004
NK cells (/ml) (ref: 150–1110)	149 (87–244) (*n* = 139)	121 (55–193) (*n* = 39)	173 (102–275) (*n* = 100)	0.011
NKT cells (/ml) (ref: 40–300)	39 (16–67) (*n* = 139)	23 (9–55) (*n* = 39)	42 (24–91) (*n* = 100)	0.001

SPO_2_: oxygen saturation; PaO_2_: partial pressure of oxygen; PaCO_2_: partial pressure of carbon dioxide; FiO_2_: fraction of inspired oxygen; IL: interleukin; CRP: C-reactive protein; PCT: procalcitonin; ESR: erythrocyte sedimentation rate; LDH: lactate dehydrogenase; ALT: alanine aminotransferase; IFN: interferon; NK: natural killer

### Association between glucocorticoids and prognosis in diabetic patients

Of the 109 patients with diabetes, 64 were treated with glucocorticoids. There were no statistically significant differences in the demographic characteristics and comorbidities between the patients treated vs. those not treated with glucocorticoids. The levels of inflammatory factors (IL-6, PCT, and CRP) in the two subgroups were also not significantly different at hospital admission. On the other hand, the CD4^+^ T lymphocytes (146 vs. 456 /ml, *p* = 0.014) and CD8^+^ T lymphocytes (81 vs. 213 /ml, *p* = 0.008) were significantly lower in patients treated with glucocorticoids compared with those who were not (Table 5). In patients with diabetes, there were no significant differences in mortality between patients treated vs. those not treated with glucocorticoids (*p* = 0.594) (Fig. 2c). Although the difference was not statistically significant, there was a 3.27-fold increase in the mortality rate of patients (> 60 years) with diabetes and critical COVID-19 treated vs. not treated with glucocorticoids (46.67% vs. 14.29%, *p* = 0.193).

**Table 5:** Clinical characteristics of diabetic patients treated or not treated by glucocorticoids

Variable	All (*n* = 109)	Glucocorticoids (*n* = 64)	No glucocorticoids (*n* = 45)	*P* value
Age (years)	74 (67–83) (*n* = 109)	75 (66–83) (*n* = 64)	74 (68–82) (*n* = 45)	0.963
Gender				
Male	64/109 (58.72)	34/64 (53.13)	30/45 (66.67)	0.157
Female	45/109 (41.28)	30/64 (46.88)	15/45 (33.33)	
Body mass index	23.42 (21.58–26.10) (*n* = 96)	24.13 (22.03–26.96) (*n* = 58)	22.42 (21.06–24.84) (*n* = 38)	0.091
In-hospital mortality	14/104 (13.46)	9/60 (15.00)	5/44 (11.36)	0.591
Smoking	24/106 (22.64)	13/61 (21.31)	11/45 (24.44)	0.703
Time from disease onset to hospitalization (d)	9 (4–12) (*n* = 109)	8 (4–11) (*n* = 64)	10 (6–13) (*n* = 45)	0.187
Clinical type				
Severe/critical	64/109 (58.72)	41/64 (64.06)	23/45 (51.11)	0.176
Mild/moderate	45/109 (41.28)	23/64 (35.94)	22/45 (48.89)	
Vaccination				> 0.999
No vaccination	49/107 (45.79)	30/62 (48.39)	19/45 (42.22)	
1 dose	9/107 (8.41)	6/62 (9.68)	3/45 (6.67)	
2 doses	17/107 (15.89)	8/62 (12.90)	9/45 (20.00)	
3 doses	32/107 (29.91)	18/62 (29.03)	14/45 (31.11)	
Comorbidities				
Cardiovascular disease	90/109 (82.57)	53/64 (82.81)	37/45 (82.22)	0.936
Chronic pulmonary disease	16/107 (14.95)	8/62 (12.90)	8/45 (17.78)	0.485
Chronic kidney disease	18/107 (16.82)	12/62 (19.35)	6/45 (13.33)	0.411
Chronic liver disease	4/107 (3.74)	2/62 (3.23)	2/45 (4.44)	> 0.999
Others	17/97 (17.53)	10/54 (18.52)	7/43 (16.28)	0.773
Vital signs on hospital admission				
Temperature (°C)	36.5 (36.3–36.8) (*n* = 106)	36.6 (36.4–36.8) (*n* = 61)	36.5 (36.3–36.8) (*n* = 45)	0.832
Respiration rate (times/min)	21 (20–22) (*n* = 106)	20 (20–22) (*n* = 61)	22 (20–23) (*n* = 45)	0.068
Heart rate (beats/min)	85 (77–98) (*n* = 105)	84 (76–92) (*n* = 60)	86 (77–102) (*n* = 45)	0.222
Systolic pressure (mmHg)	129 (115–144) (*n* = 106)	129 (114–145) (*n* = 61)	129 (117–143) (*n* = 45)	0.816
Diastolic pressure (mmHg)	75 (68–83) (*n* = 105)	75 (68–84) (*n* = 60)	75 (68–81) (*n* = 45)	0.727
Blood-gas analysis on hospital admission				
SPO_2_ (%) (ref: ≥95%)	96.0 (93.0–98.0) (*n* = 96)	95.0 (91.0–98.0) (*n* = 56)	97.0 (96.0–98.0) (*n* = 40)	0.002
pH (ref: 7.35–7.45)	7.403 (7.361–7.441) (*n* = 103)	7.410 (7.357–7.441) (*n* = 61)	7.401 (7.368–7.435) (*n* = 42)	0.658
PaO_2_ (mmHg) (ref: 75–100)	85.3 (69.3-114.2) (*n* = 102)	82.0 (68.8–107.0) (*n* = 61)	90.0 (74.0-121.6) (*n* = 41)	0.377
PaCO_2_ (mmHg) (ref: 35–45)	35.9 (32.0-39.3) (*n* = 102)	35.6 (32.0-40.2) (*n* = 61)	35.9 (32.0–39.0) (*n* = 41)	0.873
FiO_2_ (%)	21 (21–29) (*n* = 91)	21 (21–32) (*n* = 54)	21 (21–29) (*n* = 37)	0.301
HCO_3_^−^ (mmol/L) (ref: 22–32)	22.6 (19.6–24.7) (*n* = 100)	22.5 (20.0-24.7) (*n* = 60)	22.9 (18.8–24.7) (*n* = 40)	0.569
First measurements of biochemical indicators after hospitalization				
White blood cell count (10^9^/L) (ref: 4.5–11)	7.39 (5.55–11.47) (*n* = 105)	7.33 (5.10-11.96) (*n* = 61)	7.76 (6.28–10.83) (*n* = 44)	0.410
Lymphocyte count (10^9^/L) (ref: 0.6–4.4)	0.94 (0.60–1.38) (*n* = 105)	0.90 (0.60–1.30) (*n* = 61)	1.02 (0.57–1.50) (*n* = 44)	0.780
Platelet count (10^9^/L) (ref: 150–450)	208 (147–302) (*n* = 104)	199 (138–307) (*n* = 61)	226 (162–281) (*n* = 43)	0.364
Na^+^ (mmol/L) (ref: 136–145)	136.4 (134.1–139.0) (*n* = 101)	136.1 (133.4-138.6) (*n* = 59)	137.1 (134.5-139.5) (*n* = 42)	0.233
K^+^ (mmol/L) (ref: 3.7–5.2)	3.95 (3.61–4.29) (*n* = 101)	3.90 (3.59–4.30) (*n* = 59)	3.99 (3.62–4.29) (*n* = 42)	0.839
Cl^−^ (mmol/L) (ref: 96–106)	102.2 (99.4-107.9) (*n* = 101)	101.4 (98.9-107.1) (*n* = 59)	105.4 (100.2-108.9) (*n* = 42)	0.107
Blood glucose (mmol/L) (ref: 3.9–5.6)	11.47 (7.91-15.00) (*n* = 99)	11.93 (8.48–15.32) (*n* = 59)	11.13 (7.46–14.30) (*n* = 40)	0.363
D-dimer (ng/mL) (ref: <500)	920 (606–1493) (*n* = 104)	921 (552–1548) (*n* = 61)	919 (621–1378) (*n* = 43)	0.905
CRP (mg/L) (ref: <0.6)	26.55 (5.83–84.51) (*n* = 70)	28.36 (7.75–73.59) (*n* = 48)	14.75 (3.06-122.35) (*n* = 22)	0.470
PCT (ng/ml) (ref: <0.05)	0.07 (0.03–0.38) (*n* = 97)	0.08 (0.03–0.38) (*n* = 57)	0.06 (0.03–0.25) (*n* = 40)	0.454
Ferritin (ng/mL) (ref: 30–400)	910 (537–1600) (*n* = 52)	927 (600–1740) (*n* = 32)	813 (472–1289) (*n* = 20)	0.328
ESR (mm/h) (ref: <15)	64 (40–92) (*n* = 82)	57 (40–83) (*n* = 47)	76 (43–101) (*n* = 35)	0.263
LDH (U/L) (ref: 120–250)	264.1 (215.8–361.0) (*n* = 88)	281.4 (233.3-396.9) (*n* = 51)	229.1 (198.6-317.7) (*n* = 37)	0.021
Troponin/hypersensitive troponin (ng/ml) (ref: <17.5)	11.00 (5.70–19.50) (*n* = 87)	10.40 (5.70–16.10) (*n* = 49)	11.60 (6.11–22.98) (*n* = 38)	0.431
Myoglobin (ng/ml) (ref: <70)	68.4 (37.9-143.1) (*n* = 94)	60.3 (36.0-143.1) (*n* = 55)	74.5 (40.2-127.1) (*n* = 39)	0.836
Creatinine (µmol/L) (ref: 65.4-119.3)	93.2 (74.3-125.3) (*n* = 102)	91.9 (73.5-120.2) (*n* = 60)	102.9 (80.9-141.9) (*n* = 42)	0.177
Urea nitrogen (mmol/L) (ref: 3.1-8.0)	8.4 (5.5–12.4) (*n* = 101)	8.6 (5.3–12.8) (*n* = 60)	8.3 (5.7–12.2) (*n* = 41)	0.942
ALT (U/L) (ref: 9–50)	23.4 (15.9–37.2) (*n* = 87)	28.6 (16.7–39.2) (*n* = 52)	23.0 (14.1–29.0) (*n* = 35)	0.112
First measurements of cytokines and immune cell counts after hospitalization				
IL-6 (ng/L) (ref: <17.4)	25.33 (8.36–47.64) (*n* = 61)	20.05 (8.57–45.46) (*n* = 38)	26.86 (8.96–67.80) (*n* = 23)	0.618
IL-8 (ng/L) (ref: <20.6)	48.05 (27.12-144.76) (*n* = 43)	43.96 (26.26-102.26) (*n* = 28)	50.69 (29.47-158.55) (*n* = 15)	0.641
IFN-r (ng/L) (ref: <7.42)	1.36 (0.40–2.19) (*n* = 51)	1.61 (0.45–2.19) (*n* = 32)	1.17 (0.36–2.20) (*n* = 19)	0.552
CD4^+^ T cells (/ml) (ref: 550–1440)	319 (129–574) (*n* = 38)	146 (59–409) (*n* = 21)	456 (208–758) (*n* = 17)	0.014
CD8^+^ T cells (/ml) (ref: 320–1250)	141 (43–256) (*n* = 37)	81 (36–167) (*n* = 20)	213 (125–322) (*n* = 17)	0.008
NK cells (/ml) (ref: 150–1110)	121 (55–193) (*n* = 39)	148 (49–207) (*n* = 23)	120 (61–186) (*n* = 16)	0.689
NKT cells (/ml) (ref: 40–300)	23 (9–55) (*n* = 39)	27 (10–48) (*n* = 23)	19 (9–60) (*n* = 16)	0.977

SPO_2_: oxygen saturation; PaO_2_: partial pressure of oxygen; PaCO_2_: partial pressure of carbon dioxide; FiO_2_: fraction of inspired oxygen; IL: interleukin; CRP: C-reactive protein; PCT: procalcitonin; ESR: erythrocyte sedimentation rate; LDH: lactate dehydrogenase; ALT: alanine aminotransferase; IFN: interferon; NK: natural killer

## Discussion

The proportion of patients with severe Omicron variant COVID-19 was higher in the diabetic group than in the non-diabetic group. The diabetic patients showed hyperinflammation and low T cell counts compared with non-diabetic patients. In patients with the Omicron variant COVID-19, the mortality of patients with diabetes was higher than among non-diabetic patients. Among diabetic patients, glucocorticoid treatment did not influence the mortality rate. The findings suggest that caution should be taken when treating diabetic patients with glucocorticoids after infection with severe/critical SARS-CoV-2.

According to early data on COVID-19 cases reported by the USA Centers for Disease Control and Prevention (CDC), diabetes is the most common comorbidity among patients with COVID-19, accounting for 10.9% of all cases [22]. The summary of 72,314 patients with COVID-19 by the Chinese CDC showed that the mortality rate of diabetic patients (7.3%) was only second to the mortality of patients with cardiovascular disease (10.5%), while the mortality rate in the general population of patients with COVID-19 was 2.3% [23]. Meta-analyses also showed that compared with non-diabetic patients, diabetes was associated with an increased mortality rate among patients with COVID-19 [24, 25]. Therefore, in patients with diabetes and COVID-19, the risk of progression to severe disease should be considered, and early interventions should be performed, even in the presence of the Omicron variant of SARS-CoV-2.

Several factors could explain the higher mortality observed in diabetic patients. Indeed, diabetes is a systemic disease often associated with comorbidities like obesity, cardiovascular diseases, peripheral neuropathy, and diabetic nephropathy [26]. In this study, the percentage of patients with comorbidities other than diabetes (i.e., cardiovascular disease and neurological disease) was higher in diabetic patients than in non-diabetic patients, which could be associated with the poor COVID-19 prognosis of patients with diabetes [27]. Indeed, these comorbidities are associated with a higher risk of poor outcomes in patients with COVID-19 and could contribute to higher mortality [23, 28–30].

The present study also showed that after infection with the Omicron variant of SARS-CoV-2, inflammatory factors (e.g., IL-6, CRP, and PCT) were increased significantly in diabetic patients compared with non-diabetic patients, suggesting that diabetic patients might be more likely to have a hyperinflammatory response to COVID-19. Diabetes is associated with significant low-grade inflammation [10, 11], and COVID-19 could have an additive or synergistic effect on inflammation in diabetic patients. Guo et al. [8] showed that the levels of IL-6 and CRP were elevated significantly in diabetic patients with COVID-19, leading to a pro-inflammatory state that could promote systemic inflammation in COVID-19 patients. Therefore, the pro-inflammatory state associated with diabetes could contribute to a higher mortality rate in patients with diabetes.

In addition to increased inflammation, the present study also found that the T cells (CD4^+^ T lymphocytes, CD8^+^ T lymphocytes, NK cells, and NKT cells) in patients with diabetes were significantly decreased. A previous study showed T cell function impairment, CD4^+^ T cell reduction, and pathogen-specific memory Th17 cell response impairment to *Streptococcus pneumonia* in diabetic patients [31]. Kumar et al. [32, 33] investigated the functions of CD8^+^ T cells and NK cells and found that the production of cytokines (IFN-γ, IL-2, IL-17 A/F, and TNF-α) and the expression of cytotoxic molecules (perforin, granzyme B, and CD107a) were reduced in diabetic patients. A cross-sectional study showed that diabetic patients with severe COVID-19 had the lowest lymphocyte counts, indicating the presence of a low host immune response and a high risk of bacterial infections that could lead to aggravated organ damage [34]. In addition to the reduction of cellular immunity, the reduction of B cells (CD19^+^) could also reduce or delay the production of antibodies, thus delaying viral clearance and leading to worse outcomes [34]. The decreased immune cell counts in patients with diabetes and COVID-19 could contribute to a poorer prognosis of COVID-19 [35, 36].

Glucocorticoids are an effective method for suppressing the inflammatory response [12, 37]. Previous studies on COVID-19 showed that glucocorticoids could inhibit the inflammatory storm and improve the prognosis of COVID-19 patients [12, 37]. Thus, glucocorticoids have become a recommended treatment for patients with severe COVID-19. Still, glucocorticoids decrease T cell functions and could lead to adverse consequences (such as secondary infection and delayed viral clearance) in patients with low T cell counts, such as diabetic patients with COVID-19, affecting the patient outcomes. Still, there were no significant differences in age, BMI, and comorbidities between patients treated or not with glucocorticoids, and the two groups were still comparable. In addition, this study could not analyze the dynamic changes in T cells because of data availability. A meta-analysis by the WHO Rapid Evidence Appraisal for COVID-19 Therapies (REACT) Working Group suggests that systemic glucocorticoids could decrease the mortality of patients with critical COVID-19 [15], supported by a network meta-analysis of 48 trials [38]. Still, glucocorticoids are not included in the treatment guidelines for COVID-19, and giving glucocorticoids to patients with COVID-19 should be based on the severity of the disease and a careful consideration of the potential benefits and harms [39–41]. The present study suggests that patients with diabetes represent a special population of patients with COVID-19 in whom the use of glucocorticoids would warrant additional consideration.

Glucocorticoids can exacerbate blood glucose abnormalities and induce complications like hyperglycemia, favoring the spread of pathogens and kidney injuries [19, 42], while diabetes and hyperglycemia are independent predictors of mortality and incidence in patients with ARDS [43]. Although the blood glucose differences were not statistically different between the patients treated with or without glucocorticoids in the diabetic group, the differences tended to be higher in those treated with glucocorticoids, suggesting that the impact of glucocorticoid therapy on blood glucose fluctuations and prognosis should be considered. Glucocorticoids are the most common reason for developing potentially life-threatening in-hospital hyperglycemic hyperosmolar syndrome (HHS) in diabetic patients [44]. Still, the study was retrospective, and continuous glucose data during hospitalization were not available.

This study was not without limitations. Firstly, the investigation did not delve into the relationship between timing, dose, duration, and prognosis post-glucocorticoid therapy. Secondly, reasons for glucocorticoid use were unclear, likely based on physician experience as per Chinese guidelines. Thirdly, glucose levels and T cell counts in diabetic patients were not continuously monitored, impacting prognosis assessment. Finally, data gaps included background medication details, diabetes diagnosis specifics, nucleic acid test trends, SARS-CoV-2 variant identification, comorbidities in non-diabetic patients, and time data for Cox analysis.

## Conclusions

Diabetic patients have a higher incidence of severe/critical COVID-19 after infection with Omicron SARS-CoV-2, showing manifestations of hyperinflammation and T cell dysfunction. Among the patients with Omicron variant COVID-19, those with diabetes had higher mortality than those without comorbidities. Among patients with diabetes, the use of glucocorticoids did not influence mortality. Nevertheless, the findings indicate that glucocorticoids should be used cautiously in diabetic patients with severe/critical COVID-19 caused by the Omicron variant.

### Electronic supplementary material

Below is the link to the electronic supplementary material.

Supplementary Material 1

## Data Availability

All data generated or analyzed during this study are included in this article.

## Abbreviations

COVID 19: Coronavirus disease 2019
WHO: World Health Organization
RT-PCR: Reverse transcription polymerase chain reaction
BMI: Body mass index
IQR: Interquartile range
CRRT: Continuous renal replacement therapy
CDC: Control and Prevention
HHS: Hyperglycemic hyperosmolar syndrome
