# Supplementary material for: Impact of diabetes on COVID-19 and glucocorticoids on patients with COVID-19 and diabetes during the Omicron variant epidemic: a multicenter retrospective cohort study in South China
Source: BMC Infect Dis. 2024 Jul 18;24:706. doi: 10.1186/s12879-024-09287-z (PMC11256567; doi:10.1186/s12879-024-09287-z)
Supplement: Supplementary file 1 — Supplementary Material 1 [file 12879_2024_9287_MOESM1_ESM.docx]

**Table S1** Disease severity classification and incidence of clinical symptoms.

| **Disease Severity** | **All （n=400, 100%）** | **Mild （n=58, 14.50%）** | **Moderate （n=164, 41.00%）** | **Severe （n=75, 18.75%）** | **Critical （n=103, 25.75%）** |
| --- | --- | --- | --- | --- | --- |
| Age (years) | 71 (60-81) (n=400) | 65 (58-73) (n=58) | 69 (59-78) (n=164) | 75 (69-84) (n=75) | 73 (64-83) (n=103) |
| Gender |  |  |  |  |  |
| Male | 244/400 (61.00) | 32/58 (55.17) | 96/164 (58.54) | 49/75 (65.33) | 67/103 (65.05) |
| Female | 156/400 (39.00) | 26/58 (44.83) | 68/164 (41.46) | 26/75 (34.67) | 36/103 (34.95) |
| BMI | 23.08 (20.43-25.39) (n=340) | 21.37 (19.10-23.69) (n=55) | 23.35 (21.41-25.65) (n=146) | 23.88 (22.00-26.26) (n=65) | 22.63 (19.67-25.23) (n=74) |
| Smoking history | 96/391 (24.55) | 20/58 (34.48) | 40/164 (24.39) | 10/75 (13.33) | 26/94 (27.66) |
| Vaccination |  |  |  |  |  |
| Not vaccinated | 154/383 (40.21) | 18/58 (31.03) | 60/164 (36.59) | 37/75 (49.33) | 39/86 (45.35) |
| 1 dose | 23/383 (6.01) | 1/58 (1.72) | 9/164 (5.49) | 4/75 (5.33) | 9/86 (10.47) |
| 2 doses | 58/383 (15.14) | 8/58 (13.79) | 29/164 (17.68) | 10/75 (13.33) | 11/86 (12.79) |
| 3 doses or more | 148/383 (38.64) | 31/58 (53.45) | 66/164 (40.24) | 24/75 (32.00) | 27/86 (31.40) |
| Comorbidities |  |  |  |  |  |
| Diabetic | 109/400 (27.25) | 6/58 (10.34) | 39/164 (23.78) | 33/75 (44.00) | 31/103 (30.10) |
| Cardiovascular disease | 219/400 (54.75) | 25/58 (43.10) | 77/164 (46.95) | 52/75 (69.33) | 65/103 (63.11) |
| Chronic pulmonary disease | 87/393 (22.14) | 23/58 (39.66) | 37/164 (22.56) | 14/75 (18.67) | 13/96 (13.54) |
| Chronic kidney disease | 40/393 (10.18) | 1/58 (1.72) | 14/164 (8.54) | 11/75 (14.67) | 14/96 (14.58) |
| Chronic liver disease | 16/393 (4.07) | 2/58 (3.45) | 7/164 (4.27) | 2/75 (2.67) | 5/96 (5.21) |
| Others | 85/363 (23.42) | 9/58 (15.52) | 43/164 (26.22) | 12/75 (16.00) | 21/66 (31.82) |
| At least one comorbidity | 334/393 (84.99) | 46/58 (79.31) | 135/164 (82.32) | 67/75 (89.33) | 86/96 (89.58) |
| **Symptoms** |  |  |  |  |  |
| Fever | 215/390 (55.13) | 22/58 (37.93) | 92/164 (56.10) | 45/75 (60.00) | 56/93 (60.22) |
| Cough | 345/388 (88.92) | 54/58 (93.10) | 144/164 (87.80) | 70/75 (93.33) | 77/91 (84.62) |
| Fatigue | 148/387 (38.24) | 21/58 (36.21) | 64/164 (39.02) | 28/75 (37.33) | 35/90 (38.89) |
| Nasal congestion | 20/381 (5.25) | 2/58 (3.45) | 8/164 (4.88) | 5/75 (6.67) | 5/84 (5.95) |
| Running nose | 23/381 (6.04) | 5/58 (8.62) | 6/164 (3.66) | 7/75 (9.33) | 5/84 (5.95) |
| Myalgia or arthralgia | 39/379 (10.29) | 5/58 (8.62) | 17/164 (10.37) | 8/75 (10.67) | 9/82 (10.98) |
| Hypogeusia | 2/378 (0.53) | 1/58 (1.72) | 1/164 (0.61) | 0/75 (0.00) | 0/81 (0.00) |
| Palpitation | 17/382 (4.45) | 1/58 (1.72) | 7/164 (4.27) | 2/75 (2.67) | 7/85 (8.24) |
| Shortness of breath | 219/386 (56.74) | 27/58 (46.55) | 91/164 (55.49) | 44/75 (58.67) | 57/89 (64.04) |
| Hemoptysis | 13/378 (3.44) | 4/58 (6.90) | 9/164 (5.49) | 0/75 (0.00) | 0/81 (0.00) |
| Diarrhea | 15/400 (3.75) | 1/58 (1.72) | 5/164 (3.05) | 3/75 (4.00) | 6/103 (5.83) |
